# Relationship between radiation doses to heart substructures and radiation pneumonitis in patients with thymic epithelial tumors

**DOI:** 10.1038/s41598-020-68168-y

**Published:** 2020-07-07

**Authors:** Natsuo Tomita, Katsuhiro Okuda, Yasutaka Ogawa, Masato Iida, Yuta Eguchi, Yuto Kitagawa, Kaoru Uchiyama, Taiki Takaoka, Ryoichi Nakanishi, Yuta Shibamoto

**Affiliations:** 1 Departments of Radiology, Nagoya City University Graduate School of Medical Sciences, 1 Kawasumi, Mizuho-cho, Mizuho-ku, Nagoya, Aichi 467-8601 Japan; 2 Departments of Oncology, Immunology and Surgery, Nagoya City University Graduate School of Medical Sciences, 1 Kawasumi, Mizuho-cho, Mizuho-ku, Nagoya, Aichi 467-8601 Japan; 3 Department of Radiology, Kariya Toyota General Hospital, 5-15 Sumiyoshi-cho, Kariya, Aichi 448-8505 Japan; 4 Narita Memorial Proton Center, 78 Shirakawa-cho, Toyohashi, Aichi 441-8021 Japan

**Keywords:** Oncology, Cancer, Outcomes research, Risk factors

## Abstract

Radiation doses to the heart are potentially high in patients undergoing radiotherapy for thymoma or thymic carcinoma because of their origin site and propensity for pericardial invasion. We investigated potential relationships between radiation pneumonitis (RP) and the dosimetric parameters of lung and heart substructures in patients with thymic epithelial tumors. This retrospective study included 70 consecutive patients who received definitive or postoperative radiotherapy at a median dose of 58.3 Gy. Heart substructures were delineated according to a published atlas. The primary end point of ≥ grade 2 RP was observed in 13 patients (19%) despite a low lung dose; median lung V20 (i.e. percentage of the volume receiving at least 20 Gy) was only 16.6%. In a univariate analysis, four lung parameters, heart V35, three pulmonary artery (PA) parameters, two left ventricle parameters, and left atrium V35 were associated with the development of RP. In a multivariate analysis, only PA V35 remained significant (hazard ratio 1.04; 95% CI 1.01–1.07, *p* = 0.007). PA V35 of the RP versus non-RP groups were 84.2% versus 60.0% (*p* = 0.003). The moderate dose sparing of PA could be a candidate as a planning constraint for reducing the risk of RP in thoracic radiotherapy.

## Introduction

Thymic epithelial tumors (TET) generally originate within the thymus of the anterior mediastinal region, and these advanced tumors are more likely to invade the pericardial space. When patients with advanced thymoma or thymic carcinoma receive radiotherapy (RT), doses to the heart may be markedly higher than in those with locally advanced (LA) non-small-cell lung cancer (NSCLC). The RTOG 0617 study recently reported a relationship between heart doses and overall survival (OS) in LANSCLC patients treated with chemoradiotherapy (CRT)^1^. Thus, heart V5 and V30 (i.e. the percentage of the normal structure volume receiving more than the indicated dose) were both identified as important predictors of OS^1^. Similar findings were reported by Speirs et al.^2^; the heart dose was associated with both OS and cardiac toxicity in LANSCLC patients treated with CRT. Potential relationships between doses to heart substructures and non-cancer death have also been investigated in early-stage NSCLC patients treated with stereotactic body radiation therapy (SBRT)^3^. However, these studies left one shared question unanswered: the uncertainty of the cause of death. Difficulties are often associated with distinguishing among death due to RT-associated cardiac toxicity, other treatment-related death, and death due to comorbidities, particularly in retrospective studies^2,3^.

Several types of RT-associated cardiac toxicities have been identified: e.g. ischemic, pericardial, valvular, and arrhythmic^4,5^. Each type of RT-associated cardiac toxicity has a different underlying mechanism^4,5^; however, tissue fibrosis is a common mediator. For example, patients with breast cancer and lymphoma are at risk of ischemic cardiac disease, which typically develops 10 years or more after RT^6–10^. On the other hand, it currently remains unclear whether the effects of the dose to the heart are confined to the heart itself. Huang et al.^11^ investigated the impact of the heart dose on the incidence of radiation pneumonitis (RP) in LANSCLC patients treated with definitive RT. They reported that heart V65 was more strongly associated with the development of RP than lung parameters^11^. Thus, it has not yet been clarified whether severe RP is a cause of non-cancer death^1–3^.

In TET patients treated consistently with definitive or postoperative RT^12^, RP often develops despite a lower mean lung dose (MLD) and lung V20, which is the most important predictor for the development of symptomatic RP in LANSCLC patients^13,14^. Against this background, we hypothesized that doses to the heart or heart substructures are associated with the development of RP in TET patients. In the present study, we examined the incidence of RP and dosimetric parameters of the heart, heart substructures, and lungs in TET patients, and investigated potential relationships between these dosimetric parameters and RP.

## Methods

### Patient characteristics

The present study was performed after approval by the Institutional Review Board of Nagoya City University Graduate School of Medical Sciences (Approval Number: 60-19-0177). Since this study was a retrospective observational study, the Nagoya City University Ethics Committee waived the need for informed consent as part of the study approval in line with the Ethical Guidelines for Medical and Health Research Involving Human Subjects in Japan. Therefore, research content was disclosed in the form of opt-out on the website. We identified 70 consecutive TET patients who received definitive (n = 24) or postoperative (n = 46) RT between 2004 and 2017 at our single institution. All patients consented to the treatment. All patients had histologically confirmed thymoma or thymic carcinoma. Data on age, sex, smoking history, surgery, RT, and the use of chemotherapy and steroid therapy were collected. The present study followed the ethical standards laid down in the 1964 Declaration of Helsinki and its later amendments. Patient characteristics are summarized in Table 1. Median age was 61 years. Tumor types were thymoma in 41 patients (59%) and thymic carcinoma in 29 (41%).

**Table 1:** Patient and treatment characteristics.

Characteristic	*n* = 70
Age (years)	61 (29–83)
Sex male/female	34 (49%)/36 (51%)
Smoking history^a^	16 (23%)
**Histology**	
Thymoma	41 (59%)
A/AB/B1/B2/B3^b^	4/1/4/18/11/3
Thymic carcinoma	29 (41%)
SCC/LCNEC/adenocarcinoma	26/2/1
**Stage**	
T1/2/3/4	2 (3%)/8 (11%)/28 (40%)/23 (33%)
Local recurrence	9 (13%)
N0/1/2	55 (78%)/6 (9%)/9 (13%)
M0/1a/1b	49 (21%)/14 (10%)/7 (10%)
Surgery	46 (66%)
Chemotherapy use	34 (49%)
Steroid therapy use	21 (30%)
**RT technique**	
3DCRT/IMRT	66 (94%)/4 (6%)
**RT dose (Gy)**	
Median total dose	58.3 (15.0–70.0)
Median fraction dose	2.0 (1.5–2.5)
Median EQD2 dose	56.9 (13.5–70.0)
Overall treatment time (days)	43 (13–112)
GTV volume (cc)	69.5 (2.0–1564)
Lung volume (cc)	2,228 (1,110–5,246)
Lung V20 (%)	16.6 (0–48.9)
Mean lung dose (Gy)	9.7 (2.0–24.7)
Mean heart dose (Gy)	14.9 (0.4–44.9)
Mean left atrium dose (Gy)	17.8 (0.3–49.1)
Mean left ventricle dose (Gy)	2.1 (0.1–39.7)
Mean right atrium dose (Gy)	6.1 (0.1–56.5)
Mean right ventricle dose (Gy)	4.6 (0.2–41.7)
Mean ascending aorta dose (Gy)	37.9 (0.6–67.3)
Mean pulmonary artery dose (Gy)	40.8 (0.9–60.4)

Data are shown as n (%) or medians (range). Data were described as equivalent 2-Gy fractions (EQD2) using the LQ model with α/β = 3 Gy outside the median total dose. The tumor bed was regarded as GTV for postoperative cases.

*SCC* squamous cell carcinoma, *LCNEC* large cell neuroendocrine carcinoma, *RT* radiotherapy, *3DCRT* three-dimensional conformal RT, *IMRT* intensity-modulated radiation therapy, *EQD2* equivalent 2-Gy fractions, *GTV* gross tumor volume, *V20* percentage volume receiving at least 20 Gy.

^a^Missing in 9 patients. ^b^World Health Organization (WHO) classification, missing in 3 patients.

### RT and chemotherapy

**Figure 1:**
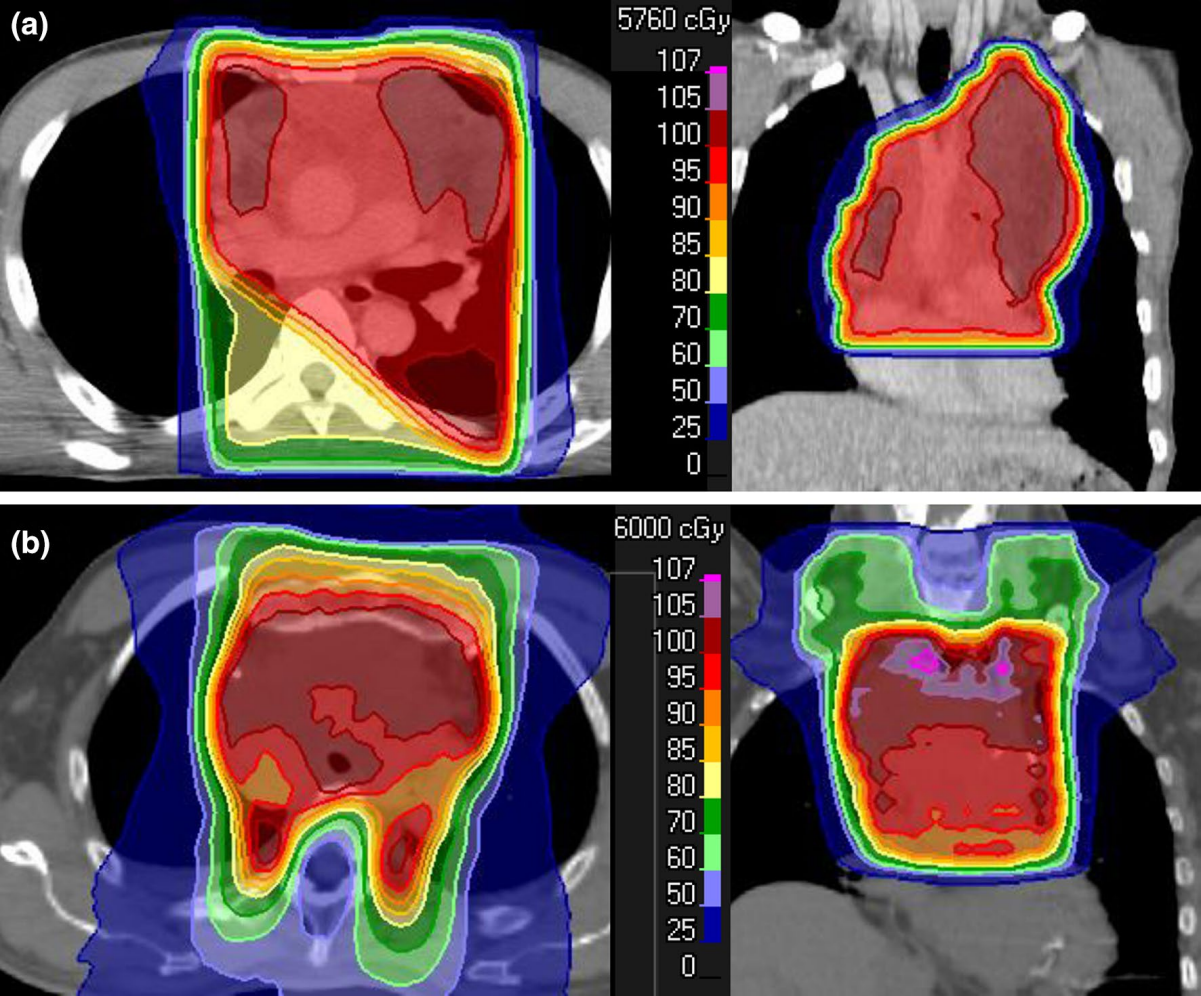
Examples of dose distributions in patients with thymic epithelial tumors treated with three-dimensional conformal RT (3DCRT) (**a**) or image-guided intensity-modulated radiation therapy (IG-IMRT) (**b**).

All patients were immobilized in a supine position and underwent computed tomography (CT) with a 2.5- or 3.2-mm slice thickness. The gross tumor volume (GTV) was defined as all known gross disease based on CT, magnetic resonance imaging, and/or ^18^F-fluorodeoxyglucose positron emission tomography. The clinical target volume (CTV) included the GTV or tumor bed with an additional margin of 1.0–1.5 cm in principle. The entire hemithorax was also included in the CTV only in 6 patients (9%) with highly-suspected microscopic residuals after the surgical resection of disseminated lesions. The planning target volume (PTV) was defined as the CTV plus a 0.5-cm margin in principle. Most patients (n = 66, 94%) were treated with three-dimensional conformal RT (3DCRT) without any image guidance system. Image-guided intensity-modulated radiation therapy (IG-IMRT) was employed to treat another 4 patients (6%). Dose distributions were calculated on a 3DCRT planning system: Eclipse (Varian Medical Systems, Palo Alto, USA) for patients treated with 3DCRT and the Tomoprovider Radiation Treatment System (Tomotherapy, Madison, USA) for patients treated with IMRT. The superposition algorithm was used for plan calculations of 3DCRT and IMRT. All patients were treated using 6–10-MV photon beams. RT was delivered to the PTV at a median dose of 58.3 Gy (range 15.0–70.0). Various 3DCRT beam arrangements were used and a commonly employed method was AP-opposed fields followed by off-cord oblique fields with the direction shown in Fig. 1a. An example of the dose distribution of IG-IMRT is shown in Fig. 1b. The median fractional dose was 2.0 Gy (range 1.5–2.5). The median dose in equivalent 2-Gy fractions (EQD2) using the LQ model with α/β = 3 Gy was 56.9 Gy (range 13.5–70.0). All doses in the present study were hereafter expressed as EQD2. RT of the entire hemithorax^12^ was performed at a median dose of 13.8 Gy (range 13.5–14.4) in 6 patients (9%), as described above.

Chemotherapy was administered in combination with RT to 34 patients (49%). Most patients (n = 31) received a platinum/taxol doublet, two received platinum/non-taxol, and one received taxol alone. High-dose steroid therapy was administered to 21 patients (30%) mainly as induction therapy for surgery or RT for advanced thymoma^15^. Most patients administered steroid therapy had T3 or T4 thymoma with WHO type AB, B1, and B2^15^.

### Dosimetric parameter analysis

All plan data were imported into the RayStation treatment planning system (RaySearch Medical Laboratories AB, Stockholm, Sweden). The heart, heart substructures, and lungs were recontoured as organs at risk (OARs) according to the published atlas^16^, and only their dose-volume data were recalculated according to the outlines of modified OARs using the RayStation. Delineation of the heart substructures was supervised by an expert radiologist in thoracic imaging and was performed by a single experienced physician. To consolidate variable fractionation schemes, biologically effective corrections to EQD2 were performed as described above. V5, V20, V35, V50, V55, and the mean dose to the lungs, heart, ascending aorta (AA), pulmonary artery (PA), left atrium (LA), left ventricle (LV), right atrium (RA), and right ventricle (RV) were used in the dosimetric parameter analysis.

### Statistical analysis

A medical interview and physical examination were performed at least every 3 months after treatment, and chest CT was obtained every 3–6 months. Major toxicities such as RP and its grade were prospectively examined in each follow-up. Follow-up times were calculated from the start date of RT. The primary endpoint was the development of ≥ grade 2 RP evaluated with CTCAE ver. 4. The OS and cancer-specific survival (CSS) were calculated by the Kaplan–Meier method and toxicity was calculated using the cumulative incidence method. The Log-rank test was used to compare OS and CSS. Fine-Gray proportional hazards models were used in univariate and multivariate analyses. Dosimetric parameters showing a correlation with RP in the univariate analysis were nominated as covariates in the multivariate analysis. Patients with missing values for covariates considered in the multivariate analysis were assumed to be missing at random. Thus, the available case analysis that uses all available data to estimate parameters of the model was performed. Correlations between each nominated parameter from RP were assessed using Spearman’s rank correlation coefficient. Pearson’s correlation coefficients were also calculated for each pair of dosimetric parameters. A correlation coefficient (CC) > 0.70 indicated a strong relationship^17^. The following variables were included in the multivariate analysis as covariates: age, sex, smoking history, combination with thoracic surgery, the use of chemotherapy, and use of steroid therapy. Welch’s t-test was also used in comparisons of dosimetric parameters between the non-RP (i.e. ≤ grade 1) and RP (i.e. ≥ grade 2) groups. All statistical analyses were performed using EZR^18^, which is a graphical user interface for R (version 3.4.1; R Foundation for Statistical Computing, Vienna, Austria). A *p* value of < 0.05 was defined as significant.

## Results

### Outcomes and toxicity

At the time of this analysis, 42 patients (60%) were alive. The median (range) follow-up time was 68 months (8–182) for all patients and 78 months (8–182) for alive patients. The 5-year OS rate was 74% (95% confidence interval [CI], 61–83). The 5-year OS rates of thymoma patients and patients with thymic carcinoma were 82% (95% CI 65–91) and 62% (95% CI 40–77), respectively (*p* = 0.11). One thymoma patient died of another disease (acute myelocytic leukemia). The 5-year CSS was 75% (95% CI 63–84). The 5-year CSS rates of thymoma patients and patients with thymic carcinoma were 85% (95% CI 69–93) and 62% (95% CI 40–77), respectively (*p* = 0.075).

**Figure 2:**
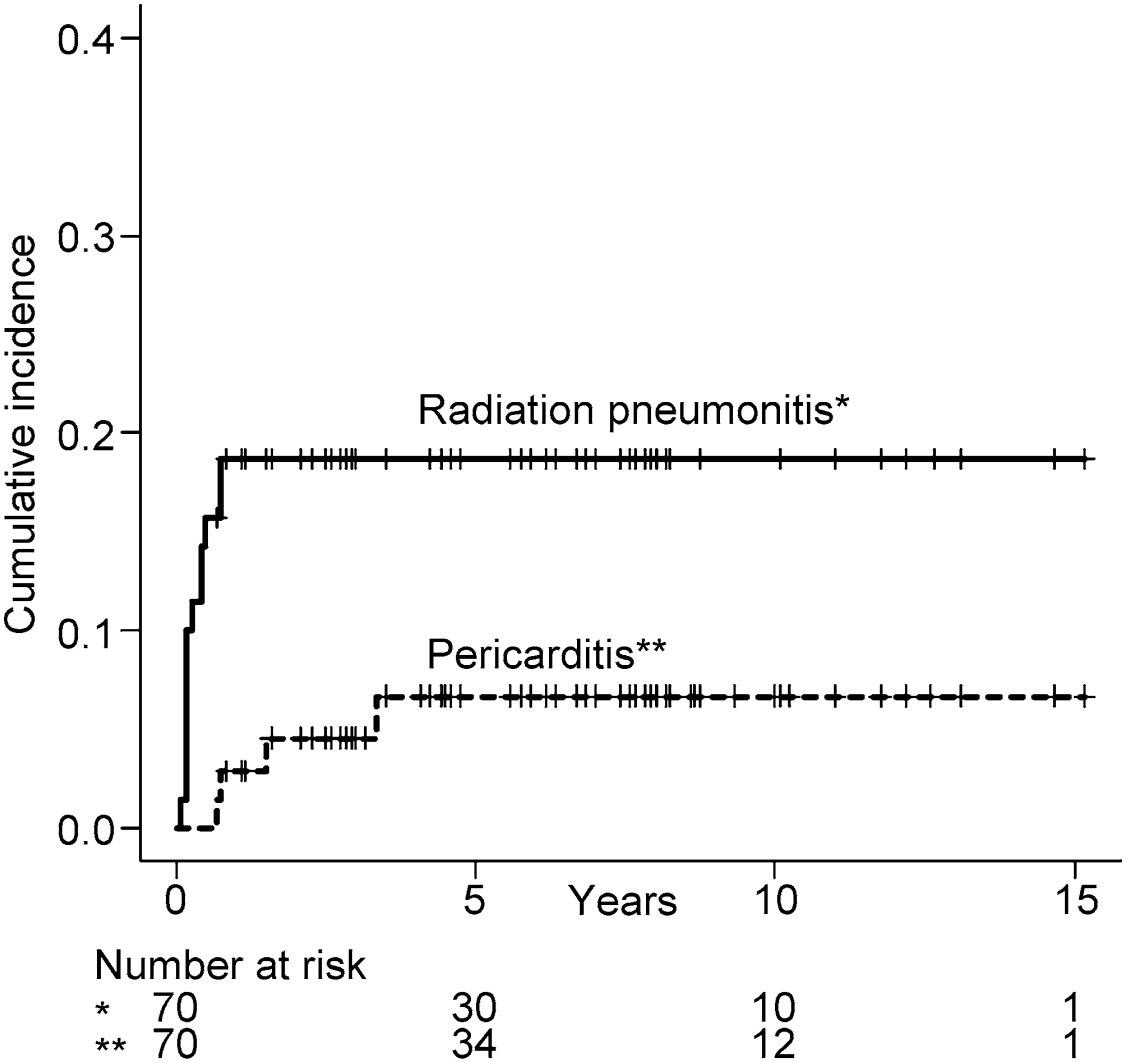
Cumulative incidence of radiation pneumonitis and cardiac toxicity among patients with thymic epithelial tumors after radiotherapy.

Thirteen patients (19%) developed ≥ grade 2 RP at a median of 2 months (range 1–9) after RT. Grade 2, 3, 4, and 5 RP developed in 9 (13%), 3 (4%), 0, and 1 (1%) patients, respectively. One patient died of RP (i.e. grade 5) 2 months after RT. Four patients (6%) developed ≥ grade 2 cardiac toxicity at a median of 14 months (range 8–40) after RT. All cardiac toxicities were pericarditis. Grade 2, 3, 4, and 5 pericarditis developed in 0, 3 (4%), 0, and 1 (1%) patients, respectively. One patient died of pericarditis (i.e. grade 5) 40 months after RT. The cumulative incidence of RP and pericarditis is shown in Fig. 2. Three patients (4%) developed both pericarditis and RP. The 5-year cumulative incidences of RP and pericarditis were 19% (95% CI 9–27) and 6.7% (95% CI 0–13), respectively. Toxicities and main dosimetric parameters are summarized in Table 2.

**Table 2:** Summary of toxicities among patients with thymic epithelial tumors after radiotherapy.

No	Age	Sex	Event	Time to event (months)	Details	MLD (Gy)	Lung V20 (%)	MHD (Gy)	PA V35 (Gy)
1	58	F	G2 RP	9	Symptomatic; medication required	12.3	21.8	8.5	75.4
2	59	F	G2 RP	2	Symptomatic; medication required	13.9	20.8	18.4	99.6
3	44	F	G2 RP	9	Symptomatic; medication required	11.8	21.6	29.4	94.1
4	55	F	G2 RP	5	Symptomatic; medication required	8.3	11.4	10.3	69.5
5	50	F	G2 RP	3	Symptomatic; medication required	16.7	35.3	44.9	100
6	72	M	G2 RP	5	Symptomatic; medication required	11.4	22.8	5.0	32.3
7	63	M	G2 RP	2	Symptomatic; medication required	5.5	9.6	14.2	94.7
8	59	F	G2 RP	2	Symptomatic; medication required	17.2	37.0	33.6	100
			G3 Carditis	18	Constrictive pericarditis; pericardiotomy required				
9	57	F	G2 RP	2	Symptomatic; medication required	16.3	36.6	39.8	100
			G5 Carditis	40	Heart failure due to constrictive pericarditis				
10	66	M	G3 RP	1	Hospitalization required	8.5	12.9	9.3	53.5
11	70	F	G3 RP	6	Hospitalization required	12.1	26.1	18.2	85.4
12	82	M	G3 RP	2	Hospitalization required	16.3	27.7	34.3	89.9
			G3 Carditis	8	Constrictive pericarditis and effusion; conservatively managed				
13	76	M	G5 RP	2	Respiratory failure due to refractory RP	17.0	33.7	14.6	100
14	56	F	G3 Carditis	9	Constrictive pericarditis; pericardiotomy required	15.3	33.0	40.7	100

Data were described as equivalent 2-Gy fractions (EQD2) using the LQ model with α/β = 3 Gy.

*MLD* mean lung dose, *V20* percentage volume receiving at least 20 Gy, *MHD* mean heart dose, *G* grade, *RP* radiation pneumonitis, *PA* pulmonary artery, *V35* percentage volume receiving at least 35 Gy.

### Dosimetric data and analysis

The median (range) values of MLD, lung V20, and heart dose (MHD) were 9.7 Gy (2.0–24.7), 16.6% (0–48.9), and 14.9 Gy (0.4–44.9), respectively. MLD and lung V20 were lower in the non-RP group than in the RP group (MLD, 9.8 vs 12.9 Gy, *p* = 0.022; lung V20, 17.4% vs 24.4%, *p* = 0.028). MHD was not different between the non-RP and RP groups (16.5 vs 21.6 Gy, *p* = 0.22).

In the univariable analysis, heart V35 (*p* = 0.016), LA V35 (*p* = 0.036), PA V20, V35, and the mean PA dose (*p* = 0.043, 0.029, and 0.026, respectively), LV V35, the mean LV dose (both *p* = 0.001), and lung V5, V20, V35, and MLD (*p* = 0.026, 0.020, 0.008, and 0.021, respectively) correlated with the development of ≥ grade 2 RP (Supplementary Table S1 online).

The relationships between significant dosimetric parameters in the univariate analysis and RP were assessed with Spearman’s rank correlation coefficients to select covariates for the multivariate analysis (Supplementary Table S2 online). As dosimetric parameters with the highest CC and lowest *p*-value for each substructure, lung V35, PA V35, LA V35, LV V35, and heart V35 were selected as potentially significant dosimetric parameters per substructure.

Correlations between two variables of the nominated per substructure parameters were assessed with Pearson’s product-moment correlation coefficients (Supplementary Table S3 online). Heart V35 was excluded from covariates in the multivariate analysis because it strongly correlated (i.e., CC > 0.70) with lung V35, LA V35, and LV V35.

The results of the multivariate analysis, in which potentially influencing clinical factors were assessed, are shown in Table 3. PA V35 remained significant (hazard ratio [HR] 1.04; 95% CI 1.01–1.07, *p* = 0.007). Age and combination steroid therapy correlated with the development of ≥ grade 2 RP (age, HR 1.06; 95% CI 1.01–1.11, *p* = 0.022; steroid therapy, HR 5.69; 95% CI 1.27–25.4, *p* = 0.023). The effects of heart V35 on the development of RP were also examined in the multivariate analysis, in which heart V35, lung V35, and potentially influencing clinical factors were included as covariates (Supplementary Table S4 online). Only steroid therapy correlated with the development of RP.

**Table 3:** Multivariate analysis of clinical factors and dosimetric parameters predicting ≥ grade 2 radiation pneumonitis among patients with thymic epithelial tumors after radiotherapy.

	HR (95% CI)	*p* value
Age (continuous)	1.06 (1.01–1.11)	0.022
Sex	0.99 (0.13–7.84)	1.00
Smoking history	1.77 (0.22–14.4)	0.59
Surgery	1.01 (0.24–4.19)	0.99
Chemotherapy	0.54 (0.13–2.19)	0.39
Steroid therapy	5.69 (1.27–25.4)	0.023
Lung V35	1.07 (0.97–1.16)	0.17
PA V35	1.04 (1.01–1.07)	0.007
LA V35	0.97 (0.93–1.02)	0.22
LV V35	1.02 (0.99–1.06)	0.15

*HR* hazard ratio, *95% CI* 95% confidence interval, The *V35* volume (%) of each structure receiving at least 35 Gy, *PA* pulmonary artery, *LV* left ventricle, *LA* left atrium.

**Figure 3:**
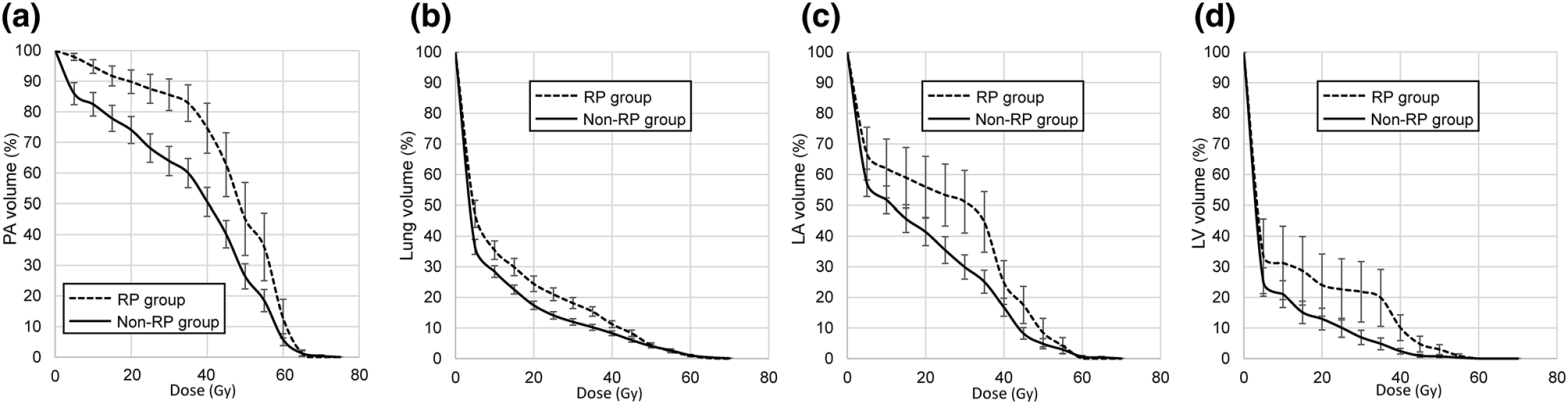
**a**–**d** Comparisons of dose-volume histograms of the pulmonary artery (PA) (**a**), lung (**b**), left atrium (LA) (**c**), and left ventricle (LV) (**d**) between ≤ grade 1 radiation pneumonitis (RP) and ≥ grade 2 RP groups. Bars represent standard errors at each dose. Data were described as equivalent 2-Gy fractions (EQD2) using the LQ model with α/β = 3 Gy.

Comparisons of the dose-volume histogram of PA, lung, LA, and LV between the non-RP and RP groups are shown in Fig. 3a–d, respectively. PA V5, V20, and V35 as well as the mean PA dose were lower in the non-RP group than in the RP group (PA V5, 86.0% vs 98.1%, *p* = 0.002; PA V20, 74.1% vs 90.6%, *p* = 0.007; PA V35, 60.0% vs 84.2%, *p* = 0.003; mean PA dose, 36.0% vs 46.4%, *p* = 0.011, respectively) (Supplementary Table S5 online). Heart parameters (V5, V20, V35, V50, and V55) did not significantly differ between the non-RP and RP groups. RA V50 and V55 were significantly higher in the non-RP group than in the RP group (RA V50, 6.7% vs 1.3%, *p* = 0.038; RA V55, 5.4% vs 0.4%, *p* = 0.027). MLD, lung V20, and lung V35 were lower in the non-RP group than in the RP group (*p* = 0.022, 0.028, and 0.012, respectively). Lung V5 was slightly lower in the non-RP group than in the RP group (*p* = 0.052), whereas lung V50 and V55 were similar between the two groups.

## Discussion

In the present study on 70 TET patients treated with RT, the univariate analysis revealed that some dosimetric parameters of the lung and heart substructures correlated with the development of RP, and the percentage of PA volume receiving at least 35 Gy (i.e. PA V35) showed the strongest correlation in the multivariate analysis. PA V35 was higher in the RP group than in the non-RP group (84.2% vs 60.0%, *p* = 0.003). This result suggests that the moderate dose sparing of PA minimizes the risk of RP in mediastinal RT, and may be applied to thoracic RT in LANSCLC patients.

A summary of studies that reported relationships between dosimetric parameters and RP after thoracic RT is shown in Table 4^1,11,13,14,19–22^. In the present study, almost 20% of TET patients developed ≥ grade 2 RP despite the lower usage rate of combination chemotherapy and significantly lower lung dose, such as lung V20 and MLD, than those for LANSCLC patients. On the other hand, MHD in the present study was not higher than those in other studies. Therefore, we considered it important to examine doses to the heart substructures in order to clarify the potential relationship between the heart dose and RP. The present study is the first to demonstrate a relationship between doses to the heart substructures and RP in thoracic RT.

**Table 4:** Summary of studies reporting relationships between dosimetric parameters and radiation pneumonitis (RP) after thoracic radiotherapy.

References	N	Tumor	Prescribed dose (Gy)	Chemotherapy (%)	RP (%)	MLD (Gy)	Lung V20 (%)	MHD (Gy)
This study	70	TET	58.3	49	19	9.7	16.6	14.9
^1^^a^	151	LANSCLC	60	100	8.6	16.5	29.0	NA
^11^	209	LANSCLC	50–84	55	23	18.2	NA	13.9
^13^	71	LANSCLC	60	100	28.2	NA	23.0	NA
^14^	836	LANSCLC	60	100	29.8	17	30	NA
^19^	125	Esophagus ca	60	100	20.8	9.5	18.2	NA
^20^	37	Esophagus ca	60	100	35.1	16.6	34.8	NA
^21^	629	LANSCLC	63	100	42	20.1	23.0	19.2
^22^	176	LANSCLC	60–65	71	39	14.4–19.4	NA	16.0–21.3

Data were described as equivalent 2-Gy fractions (EQD2) using the LQ model with α/β = 3 Gy.

*MLD* mean lung dose, *V20* percentage volume receiving at least 20 Gy, *MHD* mean heart dose, *TET* thymic epithelial tumors, *LANSCLC* locally advanced non-small-cell lung cancer, *NA* not applicable, *Esophagus ca* esophagus cancer.

^a^Data of the 60 Gy without cetuximab group.

Previous studies^11,21,22,27,28^ investigated the impact of the heart dose on the incidence of RP in NSCLC patients treated with definitive RT. Huang et al.^11^ reported that heart V65 was more strongly associated with the development of RP than lung parameters. They created a RP risk model consisting of both heart and lung parameters: heart D10 (i.e., the minimum dose to 10% of the heart receiving the highest doses), lung D35, and the maximum lung dose^11^. This was consistent with our results showing that RP may be associated with dosimetric parameters of the heart (heart substructure). Dang et al.^22^ found a correlation between dosimetric parameters of the heart and RP in univariate analyses of 176 patients, whereas multivariate analyses did not confirm this relationship. Experimental studies using animal models revealed that heart irradiation may influence the development of radiation-induced lung injury. For example, van Luijk et al. reported significant differences in breathing rates^23,24^ and lung morphology^25^ in rats after large, single radiation doses to the lung, which depended on whether the heart was within or outside the RT field. Excluding the heart from the RT field will reduce late effects after thoracic RT because the development of RP precedes late radiation fibrosis^26^. On the other hand, the following studies noted the lack of an impact of the heart dose on the development of RP. Tucker et al.^21^ showed the lack of involvement of the heart dose on the risk of moderate or severe RP in a large cohort of 629 patients. A meta-analysis of five studies reported a risk of RP in NSCLC patients with left versus right lung involvement and found no evidence for an increased risk of RP among patients with left-sided tumors^27^. However, this finding was not demonstrated by direct measurements of heart doses. Furthermore, in one of the five studies, the risk of RP slightly increased among patients with left-sided tumors (odds ratio 2.31, *p* = 0.098)^28^.

Due to conflicting findings on the relationship between the dose to the whole heart and RP, the relationship between RP and heart substructures remains unclear. To the best of our knowledge, few studies have investigated the effects of the dose to each heart substructure, even those on RT-associated cardiac toxicities^3^. In the analysis of 803 early-stage NSCLC patients treated with SBRT, doses to the LA and superior vena cava exhibited the strongest correlation with non-cancer death after a multivariate analysis^3^. Doses to the upper region of the heart and vessels and the lower 3 cm of the bronchus had the strongest correlation with non-cancer death in early-stage NSCLC patients after SBRT. These findings suggest that dose sparing, particularly in the upper region of the heart, will improve patient outcomes. In another study on 1,101 patients treated with definitive RT^29^, a voxel-by-voxel analysis was performed to examine correlating dose in each voxel against OS. The results of analyses showed that the base of the heart was identified as a dose-sensitive region, whereas MHD, heart V5, and V30 were not significant factors associated with survival. Patients receiving more than 8.5 Gy to the base of the heart had significantly worse survival (HR 1.20, *p* < 0.001). These findings were partially consistent with the present results suggesting that the sparing of PA (i.e. upper and base region of the heart) is important for minimizing the risk of RT-associated toxicity in thoracic RT.

A pooled analysis of RP data showed that some clinical factors influenced the risk of RP: comorbidity (*p* = 0.007), an older age (*p* = 0.0001), and ongoing smoking (*p* = 0.008)^27^. Age, dosimetric parameters, and the chemotherapy regimen selected allow for the significant stratification of the risk of RP^14^. To the best of our knowledge, the relationship between the combination of steroid therapy and the development of RP has not yet been demonstrated. Larger tumors in patients administered high steroid therapy may affect the development of RP more than in other patients. However, the underlying mechanisms have not yet been elucidated.

The present study has several limitations. The number of patients was small and events were relatively limited. This may have resulted in a low statistical power. Furthermore, the retrospective nature may have precluded an accurate evaluation of toxicity. On the other hand, we considered it possible to evaluate all grade 2 or higher toxicities because all patients developing grade 2 or higher toxicities underwent all procedures for toxicity evaluations. In addition, the present results may depend on our institutional methods of treating TET, including beam arrangements and prescriptions. In Supplementary Table S5, RA V50 and V55 were significantly higher in the non-RP group than in the RP group, which may have been due to our beam arrangements (Fig. 1a). Another limitation is that we were unable to investigate potential relationships between RT-associated cardiac toxicity and dosimetric parameters of the heart and heart substructures because the number of events was limited. The lack of comorbidity data was a limitation of the present study because comorbidity is one of the clinical risk factors for RP^27^. Regarding RT-associated cardiac toxicity, pre-existing cardiac disease correlated with a nearly three-fold increase in the likelihood of developing cardiac events^30^. The correlation among the doses to the lung and each heart substructure was also one of the limitations. The possibility that reducing the PA V35 could potentially increase the doses to the other substructures and the risk of RP cannot be denied from our results.

In conclusion, we investigated potential relationships between doses to heart substructures and RP in 70 TET patients. Almost 20% of TET patients developed ≥ grade 2 RP despite the low usage rate of combination chemotherapy and significantly lower lung dose than that for LANSCLC patients. In the multivariate analysis, only PA V35 remained significant among the dosimetric parameters evaluated. The present results suggest that the moderate dose sparing of PA could be a candidate as a planning constraint for reducing the risk of RP in thoracic RT, if the doses to the lung and the other heart substructures can be kept below certain levels.

## Supplementary information

Supplementary file1

## Data Availability

The datasets generated and/or analyzed during the present study are not publicly available due to ethical reasons, but are available from the corresponding author upon reasonable request.
