# Supplementary material for: Relationship between radiation doses to heart substructures and radiation pneumonitis in patients with thymic epithelial tumors
Source: Sci Rep. 2020 Jul 7;10:11191. doi: 10.1038/s41598-020-68168-y (PMC7340766; doi:10.1038/s41598-020-68168-y)
Supplement: Supplementary file 1 — Supplementary file1 [file 41598_2020_68168_MOESM1_ESM.docx]

**Relationship between radiation doses to heart substructures and radiation pneumonitis in patients with thymic epithelial tumors**

Natsuo Tomita^1^, Katsuhiro Okuda^2^, Yasutaka Ogawa^1^, Masato Iida^1^, Yuta Eguchi^1^, Yuto Kitagawa^1^, Kaoru Uchiyama^3^, Taiki Takaoka^4^, Ryoichi Nakanishi^2^, & Yuta Shibamoto^1^

Departments of ^1^ Radiology, ^2^ Oncology, Immunology and Surgery, Nagoya City University Graduate School of Medical Sciences, 1 Kawasumi, Mizuho-cho, Mizuho-ku, Nagoya, Aichi, 467-8601, Japan

^3^ Department of Radiology, Kariya Toyota General Hospital, 5-15 Sumiyoshi-cho, Kariya, Aichi, 448-8505, Japan

^4^ Narita Memorial Proton Center, 78 Shirakawa-cho, Toyohashi, Aichi, 441-8021, Japan

Corresponding author: Natsuo Tomita, MD, PhD, Department of Radiology, Nagoya City University Graduate School of Medical Sciences, 1 Kawasumi, Mizuho-cho, Mizuho-ku, Nagoya, Aichi, 467-8601, Japan

Phone: (+81)52-853-8276; Fax: (+81)52-852-5244; E-mail: c051728@yahoo.co.jp

**Supplementary Table S1** Univariate analysis with the Fine-Gray proportional-hazards model for ≥ grade 2 radiation pneumonitis

among patients with thymoma or thymic cancer after radiotherapy

**Lung Heart Ascending aorta** **Pulmonary artery**

HR 95% CI *p*-value HR 95% CI *p*-value HR 95% CI *p* -value HR 95% CI *p*-value

V5 1.03 1.00-1.05 0.026 1.01 0.99-1.03 0.31 1.01 0.99-1.03 0.38 1.07 0.99-1.15 0.074

V20 1.06 1.01-1.11 0.020 1.01 1.00-1.03 0.14 1.01 0.99-1.03 0.24 1.03 1.00-1.05 0.043

V35 1.08 1.02-1.14 0.008 1.02 1.00-1.04 0.016 1.02 1.00-1.04 0.11 1.03 1.00-1.05 0.029

V50 1.02 0.93-1.11 0.75 1.01 0.98-1.04 0.45 1.01 1.00-1.03 0.077 1.01 1.00-1.03 0.086

V55 0.98 0.87-1.11 0.75 1.00 0.97-1.04 0.80 1.01 1.00-1.03 0.11 1.01 1.00-1.03 0.12

Mean 1.12 1.02-1.24 0.021 1.03 0.99-1.08 0.14 1.03 0.99-1.06 0.17 1.05 1.01-1.10 0.026

**Left atrium Left ventricle Right atrium Right ventricle**

HR 95% CI *p*-value HR 95% CI *p*-value HR 95% CI *p*-value HR 95% CI *p*-value

V5 1.01 0.99-1.02 0.34 1.01 0.99-1.02 0.26 1.01 0.99-1.02 0.37 1.01 0.99-1.02 0.43

V20 1.01 1.00-1.03 0.18 1.01 1.00-1.03 0.060 1.00 0.99-1.02 0.55 1.01 0.99-1.02 0.38

V35 1.02 1.00-1.03 0.036 1.02 1.01-1.04 0.001 1.01 0.99-1.02 0.41 1.01 1.00-1.03 0.065

V50 1.01 0.98-1.04 0.38 1.07 0.99-1.17 0.10 0.96 0.93-0.99 0.014 1.02 0.97-1.07 0.51

V55 1.01 0.97-1.05 0.68 1.07 0.92-1.25 0.36 0.93 0.87-0.99 0.020 1.00 0.94-1.06 0.99

Mean 1.03 0.99-1.07 0.17 1.04 1.00-1.09 0.041 1.00 0.98-1.03 0.81 1.02 0.98-1.06 0.29

Data are means ± standard deviations.

*V5, V20, V35, V50,* and *V55* volumes (%) of each structure receiving at least 5, 20, 35, 50, and 55 Gy, respectively

*HR* hazard ratio, *95% CI* 95% confidence interval

**Supplementary Table S2** Spearman’s rank correlation coefficients between radiation pneumonitis and significant dosimetric parameters in a univariate analysis of patients with thymoma or thymic cancer after radiotherapy

CC *p*-value

Lung parameter

Lung V5 0.24 0.044

Lung V20 0.27 0.025

Lung V35 0.30 0.012

Mean dose 0.29 0.015

Pulmonary artery (PA) parameter

PA V20 0.20 0.10

PA V35 0.31 0.010

Mean dose 0.26 0.029

Left ventricle (LV) parameter

LV V35 0.27 0.025

Mean dose 0.16 0.18

Left atrium (LA) parameter

LA V35 0.23 0.054

Whole heart parameter

Heart V35 0.21 0.077

*V5, V20,* and *V35* volumes (%) of each structure receiving at least 5, 20, and 35 Gy, respectively

*CC* correlation coefficients

**Supplementary Table S3** Pearson’s product-moment correlation coefficient between two variables of heart V35, lung V35, PA V35, LA V35, and LV V35 among patients with thymoma or thymic cancer after radiotherapy

Lung V35 PA V35 LA V35 LV V35

CC 95% CI CC 95% CI CC 95% CI CC 95% CI

Heart V35 0.71 0.57-0.81 0.66 0.51-0.78 0.91 0.86-0.95 0.80 0.70-0.87

Lung V35 NA 0.50 0.30-0.66 0.63 0.46-0.75 0.49 0.29-0.65

PA V35 NA NA 0.61 0.44-0.74 0.37 0.15-0.56

LA V35 NA NA NA 0.68 0.53-0.79

All *p*-values were < 0.001

The *V35* volume (%) of each structure receiving at least 35 Gy

*PA* pulmonary artery, *LA* left atrium, *LV* left ventricle, *CC* correlation coefficients, *95% CI* 95% confidence interval, *NA* not applicable

**Supplementary Table S4** Multivariate analysis of clinical factors and dosimetric

parameters other than heart substructures predicting ≥ grade 2 radiation

pneumonitis among patients with thymic epithelial tumors after radiotherapy

HR (95% CI) *p*-value

Age (continuous) 1.03 (0.99-1.08) 0.13

Sex 0.57 (0.13-2.52) 0.45

Smoking history 1.74 (0.12-25.1) 0.68

Surgery 0.84 (0.14-5.09) 0.85

Chemotherapy 0.82 (0.13-5.21) 0.84

Steroid therapy 3.77 (1.16-12.3) 0.028

Lung V35 1.05 (0.95-1.16) 0.33

Heart V35 1.01 (0.99-1.03) 0.38

*HR* hazard ratio, *95% CI* 95% confidence interval,

The *V35* volume (%) of each structure receiving at least 35 Gy

**Supplementary Table S5** Comparisons of dose-volume data between ≤ grade 1 radiation pneumonitis (RP) and ≥ grade 2 RP groups

among patients with thymoma or thymic cancer after radiotherapy

**Heart** **Lung**

All patients ≤ Grade 1 RP ≥ Grade 2 RP *p*-value All patients ≤ Grade 1 RP ≥ Grade 2 RP *p*-value

V5 47.2±22.2 45.5±28.7 54.8±28.9 0.35 38.5±18.6 36.5±18.6 47.2±15.7 0.052

V20 35.3±18.5 33.1±26.3 44.7±26.0 0.23 18.7±10.2 17.4±10.0 24.4±9.1 0.028

V35 26.8±16.0 23.9±20.8 39.5±25.5 0.11 11.2±7.2 10.2±7.2 15.4±5.6 0.012

V50 9.8±15.1 9.3±13.7 12.0±12.4 0.50 4.1±4.0 4.0±4.3 4.4±2.7 0.73

V55 7.1±15.1 7.0±12.3 7.8±9.3 0.80 2.6±3.2 2.7±3.4 2.5±2.2 0.80

Mean 17.4±9.1 16.5±10.9 21.6±12.0 0.22 10.4±4.6 9.8±4.6 12.9±3.7 0.022

**Ascending aorta Pulmonary artery** **Left atrium**

≤ Grade 1 RP ≥ Grade 2 RP *p*-value ≤ Grade 1 RP ≥ Grade 2 RP *p*-value ≤ Grade 1 RP ≥ Grade 2 RP *p*-value

V5 79.7±29.7 85.7±22.9 0.37 86.0±27.1 98.1±3.9 0.002 57.3±33.8 66.8±31.1 0.36

V20 68.1±35.0 78.4±28.4 0.22 74.1±33.2 90.6±13.1 0.007 41.4±34.0 56.0±35.7 0.21

V35 57.6±35.1 74.1±30.9 0.081 60.0±36.0 84.2±20.4 0.003 25.1±28.2 44.6±35.5 0.094

V50 23.8±29.7 41.0±36.5 0.15 26.4±31.0 44.6±39.5 0.15 4.8±12.4 8.5±16.8 0.49

V55 16.3±26.3 32.7±33.6 0.20 18.5±27.3 33.2±37.9 0.22 2.9±9.8 4.2±9.6 0.69

Mean 34.3±17.2 41.1±15.5 0.17 36.0±16.2 46.4±11.0 0.011 18.9±13.2 25.0±15.3 0.22

**Left ventricle** **Right atrium** **Right ventricle**

≤ Grade 1 RP ≥ Grade 2 RP *p*-value ≤ Grade 1 RP ≥ Grade 2 RP *p*-value ≤ Grade 1 RP ≥ Grade 2 RP *p*-value

V5 25.0±35.2 33.4±42.1 0.33 35.4±38.2 45.1±35.6 0.41 31.2±34.8 35.1±36.2 0.47

V20 12.9±26.7 24.0±35.2 0.18 24.1±34.3 29.7±33.0 0.60 20.3±30.1 22.5±29.7 0.46

V35 4.8±14.7 19.8±32.3 0.078 16.5±29.0 23.6±33.7 0.51 12.4±22.2 19.5±29.0 0.23

V50 0.7±3.2 3.0±5.6 0.23 6.7±18.4 1.3±2.0 0.038 3.6±9.0 5.9±11.7 0.59

V55 0.3±1.9 0.9±2.2 0.44 5.4±16.3 0.4±0.8 0.027 2.7±7.2 3.0±5.3 0.96

Mean 6.6±8.9 14.6±4.2 0.16 12.5±15.3 13.6±12.2 0.79 10.1±11.6 12.2±13.3 0.37

Data are means ± standard deviations

The *V5, V20, V35, V50,* and *V55* volumes (%) of each structure receiving at least 5, 20, 35, 50, and 55 Gy, respectively
